# Pitfalls of single-study external validation illustrated with a model predicting functional outcome after aneurysmal subarachnoid hemorrhage

**DOI:** 10.1186/s12874-024-02280-9

**Published:** 2024-08-08

**Authors:** Jordi de Winkel, Carolien C. H. M. Maas, Bob Roozenbeek, David van Klaveren, Hester F. Lingsma

**Affiliations:** 1 Department of Neurology, Erasmus MC University Medical Center Rotterdam, 40 Doctor Molewaterplein, P.O. Box 2040, Rotterdam, Zuid-Holland 3015 GD The Netherlands; 2 Department of Public Health, Erasmus MC University Medical Center Rotterdam, Rotterdam, Zuid-Holland The Netherlands

**Keywords:** Intracranial Aneurysm, Logistic Regression, Validation Study, Prognosis, Subarachnoid Hemorrhage

## Abstract

**Background:**

Prediction models are often externally validated with data from a single study or cohort. However, the interpretation of performance estimates obtained with single-study external validation is not as straightforward as assumed. We aimed to illustrate this by conducting a large number of external validations of a prediction model for functional outcome in subarachnoid hemorrhage (SAH) patients.

**Methods:**

We used data from the Subarachnoid Hemorrhage International Trialists (SAHIT) data repository (*n* = 11,931, 14 studies) to refit the SAHIT model for predicting a dichotomous functional outcome (favorable versus unfavorable), with the (extended) Glasgow Outcome Scale or modified Rankin Scale score, at a minimum of three months after discharge. We performed leave-one-cluster-out cross-validation to mimic the process of multiple single-study external validations. Each study represented one cluster. In each of these validations, we assessed discrimination with Harrell’s c-statistic and calibration with calibration plots, the intercepts, and the slopes. We used random effects meta-analysis to obtain the (reference) mean performance estimates and between-study heterogeneity (I^2^-statistic). The influence of case-mix variation on discriminative performance was assessed with the model-based *c*-statistic and we fitted a “membership model” to obtain a gross estimate of transportability.

**Results:**

Across 14 single-study external validations, model performance was highly variable. The mean c-statistic was 0.74 (95%CI 0.70–0.78, range 0.52–0.84, I^2^ = 0.92), the mean intercept was -0.06 (95%CI -0.37–0.24, range -1.40–0.75, I^2^ = 0.97), and the mean slope was 0.96 (95%CI 0.78–1.13, range 0.53–1.31, I^2^ = 0.90). The decrease in discriminative performance was attributable to case-mix variation, between-study heterogeneity, or a combination of both. Incidentally, we observed poor generalizability or transportability of the model.

**Conclusions:**

We demonstrate two potential pitfalls in the interpretation of model performance with single-study external validation. With single-study external validation. (1) model performance is highly variable and depends on the choice of validation data and (2) no insight is provided into generalizability or transportability of the model that is needed to guide local implementation. As such, a single single-study external validation can easily be misinterpreted and lead to a false appreciation of the clinical prediction model. Cross-validation is better equipped to address these pitfalls.

**Supplementary Information:**

The online version contains supplementary material available at 10.1186/s12874-024-02280-9.

## Background

Clinical prediction models are used to predict the probability of a disease or an outcome conditional on a set of patient characteristics. As clinical prediction models are meant to facilitate clinical decision-making, it is paramount that the prognostic estimates are valid and precise. Unreliable risk estimates could give rise to faulty decision-making and thus patient harm [1]. For example, in vascular neurology we use the PHASES score (Population, Hypertension, Age, Size, Earlier Subarachnoid Hemorrhage, Site). The PHASES score is a clinical prediction model used to predict the 5-year rupture risk in patients with an unruptured intracranial aneurysm [2]. The risk of rupture predicted by the PHASES score is balanced with the risk of intervention to determine optimal management [3]. Misclassification could result in withholding preventive aneurysm treatment in high-risk patients or unnecessary treatment-related harm in low-risk patients. To investigate accuracy and precision of the prognostic estimates, validation is performed.

Two types of validation are distinguished: internal validation and external validation. Internal validation assesses the robustness of the model and the degree of overfitting (i.e., modeling random noise within the development data). External validation is used to investigate model performance in independent data that was not involved in model development. Model performance is expressed in terms of discrimination – the ability to distinguish patients likely to experience the outcome of interest from those who are not – and calibration – the agreement between the predicted and observed risk.

External validation is usually conducted in a study with data from a single center of a certain period, from a certain geographical area, and consisting of patients selected based on specific criteria. This method is called “single-study external validation”. The model performance obtained through this type of validation is often thought to represent model performance that can be expected in the population. However, this interpretation is not as straightforward as assumed. It is highly debatable whether (a single) single-study external validation provides true insight into the validity and accuracy of the risk predictions and there are several pitfalls to consider when interpreting its results [4]. There is an alternative, hybrid, internal–external cross-validation approach available for clustered data that might better address these pitfalls. Clustered data consists of multiple sources differing in multiple dimensions (e.g., geographical areas, studies, periods, etc.). In this approach, each cluster is left out once to validate the model in to obtain cluster-specific performance estimates.

Because the number of model development and validation studies is rising exponentially accurate critical appraisal of such studies is becoming increasingly important. Here, we conducted a large number of single-study validations of a prediction model for functional outcome in aneurysmal subarachnoid hemorrhage (aSAH) patients to illustrate potential pitfalls leading to misinterpretation of model performance.

## Methods

### Study population and model development

We used data from The Subarachnoid Hemorrhage International Trialists (SAHIT) data repository, an individual participant meta-analysis registry (Supplementary Material 1). The SAHIT data repository consisted of eleven randomized controlled trials (RCTs) [5–16] and ten prospective observational hospital registries (*n*= 13,046) [17–21]. Patient enrolment took place in four continents over 30 years (1983–2012). From the SAHIT data repository, we selected studies that determined functional outcome with the (extended) Glasgow Outcome Scale ((e)GOS) or modified Rankin Scale (mRS) with a minimum follow-up of 3 months (*n* = 11,931). We excluded nine data sources (Supplementary Material 2).

The GOS was dichotomized into favorable and unfavorable (defined as a GOS 4–5 versus GOS 1–3, or eGOS 4–8 versus 1–3, respectively). The GOS ranges from one to five, with five being no symptoms and one death. The eGOS is a more detailed nine-level version of the GOS. If the GOS scores were not available we used the mRS or eGOS scores. The mRS is a 7-level scale ranging from zero, no symptoms to six, death. We dichotomized the mRS into 0–3, favorable, and 4–6, unfavorable. All functional outcome scales are commonly used in stroke research [22, 23]. Conversion algorithms were described in Supplementary Material 3. Missing data were handled by using multiple imputation by chained equations. We assumed data were missing at random [24].

We refitted the previously published SAHIT (neuroimaging) model using identical data and predictors and similar modeling strategies as in the original development study [25]. The purpose of this study was not to develop a new or better model. In the SAHIT model, age, aneurysm location, aneurysm size, World Federation of Neurological Surgeons (WFNS) grade on admission, premorbid hypertension, and CT Fisher grade were used as predictors of functional outcome [26, 27]. We dichotomized the WFNS grade into I-III and IV-V and categorized aneurysm location into anterior cerebral artery, anterior communicating artery, internal cerebral artery, middle cerebral artery, posterior communicating artery, and posterior circulation.

## Model performance

We assessed discrimination with Harrell’s *c*-statistic and the model-based *c*-statistic [28]. We compared Harrell’s *c*-statistics at single-study and cross-validation validation with the internally validated optimism-corrected Harrell’s *c*-statistic obtained via bootstrapping (benchmark). Next, to quantify to which extent the case-mix variation influences the discriminative performance at validation we evaluated the difference between Harrell’s *c*-statistic and the model-based *c*-statistic. This difference is attributable to case-mix variation. We assessed calibration graphically with calibration plots and numerically with the intercept and the slope. To facilitate understanding of the study, we have included a detailed explanation of all performance measures that are discussed in this study (Supplementary Material 4).

### Validation

We performed two types of validation to assess model performance: leave-one-cluster-out internal–external cross-validation (henceforth cross-validation) and single-study external validation. With cross-validation, each cluster (representing one study or registry) is alternatingly left out of model development to assess model performance [29]. The split in the data is non-random because existing heterogeneity between clusters is utilized (by study, geographical area, period). To mimic the process of multiple single-study external validations, we assessed model performance, obtained via cross-validation, individually in each cluster as if they were predefined external validation clusters. We assessed the performance metrics with leave-one-cluster-out cross-validation as with single-study external validation.

Next, we pooled the performance with random effects meta-analysis to obtain mean performance estimates that serve as a reference value for overall external model performance [30]. We used the I^2^-statistic to describe the degree of variability in model performance that is attributable to between-cluster heterogeneity. All statistical analyses were performed with R software (version 3.6.3, R Foundation for Statistical Computing) using the *rms* (version 6.2.0) [31], *mice* (version 3.13.0) [32], *Hmisc* (version 4.5.0) [33], *metamisc* (version 0.2.5) [34], *CalibrationCurves* (version 0.1.2) [35], *metafor* (version 3.4.0) [36], and *PredictionTools* (version 0.1.0) [37] packages. We adhered to the Transparent Reporting of a multivariable prediction model for Individual Prognosis or Diagnosis checklist (TRIPOD) statement (Supplementary Material 5) [38].

### Transportability

We assessed the relatedness of the derivation and validation clusters by fitting a logistic regression “membership model” [29]. This model predicts a patient being part of the development (0) or validation (1) cluster and includes all predictors and the outcome. A high* c*-statistic of the membership model (easy discrimination) means that the development and validation clusters are not related and a low *c*-statistic (hard discrimination) vice versa. By jointly assessing Harrell’s *c*-statistic and the *c*-statistic of the membership model we obtain a gross estimate of the generalizability and transportability of the model. Generalizability is the reproducibility in a similar population and transportability is the reproducibility in a different but related population. Transportability is important because many models will be applied to populations that differ from the original study population. To illustrate, observing a high membership model *c*-statistic and a high Harrell’s *c*-statistic means that even though there was a lot of heterogeneity between clusters the model still discriminated well.

## Results

### Baseline characteristics

In the overall SAHIT data repository, the mean age was 53 years (SD 13, Table 1), 80% of patients presented in a favorable clinical condition (WFNS grade I-III, *n* = 8869), and 38% had premorbid hypertension (*n* = 2884). In 5% of patients, no hemorrhage was detected on the CT scan (Fisher grade 1, n = 493), 21% had a Fisher grade 2 (n = 2050), 39% had a Fisher grade 3 (*n* = 3762), and 35% had a Fisher grade 4 (*n* = 3440). Most ruptured aneurysms were smaller than 13 mm (84%, *n* = 7314) and 88% had a ruptured intracranial aneurysm located in the anterior circulation (*n* = 9081). Eighty percent of patients had a favorable functional outcome at a minimum of 3-month follow-up (range 60–92%, *n* = 8537).

**Table 1:** Baseline characteristics

**Variable**	**Full cohort**^**a**^ **(*n* = 11,931)**	**Concious-1** **(*n* = 413)**	**Chicago** **(*n* = 75)**	**EPO/Statin** **(*n* = 160)**	**HHU** **(*n* = 60)**	**IHAST** **(*n* = 1000)**	**IMASH** **(*n* = 327)**	**ISAT** **(*n* = 2143)**
Age – n (%)	11,931 (100)	413 (100)	75 (100)	160 (100)	60 (100)	1000 (100)	32 (100)	2143 (100)
Year – mean (SD)	53 (13)	51 (11)	51 (16)	55 (13)	56 (10)	52 (13)	57 (13)	52 (12)
WFNS grade – n (%)	11,144 (93)	413 (100)	75 (100)	160 (100)	60 (100)	1000 (100)	327 (100)	2112 (99)
I-III	8869 (80)	313 (76)	44 (59)	112 (70)	13 (22)	1000 (100)	209 (64)	2018 (96)
IV-V	2275 (20)	100 (24)	31 (41)	48 (30)	47 (78)	0	118 (36)	94 (4)
Premorbid hypertension – n (%)	7609 (64)	413 (100)	75 (100)	NR	NR	1000 (100)	327 (100)	NR
Yes (%)	2884 (38)	172 (42)	38 (51)	NR	NR	398 (40)	198 (61)	NR
CT Fisher grade – n (%)	9745 (82)	408 (99)	75 (100)	160 (100)	60 (100)	1000 (100)	327 (100)	2129 (99)
1	493 (5)	4 (1)	5 (7)	11 (7)	0	54 (5)	2 (1)	114 (5)
2	2050 (21)	85 (21)	12 (16)	8 (5)	2 (3)	342 (34)	24 (7)	360 (17)
3	3762 (39)	35 (9)	58 (77)	17 (11)	9 (15)	474 (47)	262 (80)	902 (42)
4	3440 (35)	284 (70)	0	124 (78)	49 (82)	130 (13)	39 (12)	753 (35)
Aneurysm size – n (%)	8719 (73)	NR	NR	NR	60 (100)	996 (100)	NR	2143 (100)
< 13 mm	7314 (84)	NR	NR	NR	56 (93)	878 (88)	NR	2078 (97)
≥ 13 mm	1405 (16)	NR	NR	NR	4 (7)	118 (12)	NR	65 (3)
Aneurysm location – n (%)	9980 (90)	413 (100)	NR	143 (89)	60 (100)	999 (100)	NR	2143 (100)
ACA	1952 (20)	22 (5)	NR	0	2 (3)	37 (4)	NR	536 (25)
ACOM	2020 (20)	160 (39)	NR	51 (36)	18 (30)	354 (35)	NR	549 (26)
ICA	1962 (20)	46 (11)	NR	8 (6)	3 (5)	81 (8)	NR	492 (23)
MCA	1840 (18)	69 (17)	NR	37 (26)	21 (35)	206 (21)	NR	303 (14)
PCOM	899 (9)	66 (16)	NR	32 (22)	8 (13)	237 (24)	NR	0
Posterior	1307 (13)	50 (12)	NR	15 (11)	8 (13)	84 (8)	NR	263 (12)
Functional outcome – n (%)	10,653 (89)	413 (100)	73 (97)	160 (100)	60 (100)	1000 (100)	327 (100)	2068 (97)
Favorable	8537 (80)	325 (79)	44 (60)	101 (63)	49 (82)	871 (87)	213 (65)	1790 (87)
Unfavorable	2216 (20)	99 (21)	29 (40)	59 (37)	11 (18)	129 (13)	114 (35)	278 (13)
**Variable**	**Full cohort**^**a**^ **(*n* = 11,931)**	**Leeds** **(*n* = 117)**	**MAPS** **(*n* = 228)**	**MASH1/2** **(*n* = 1484)**	**D-SAT** **(*n* = 439)**	**SHOP** **(*n* = 1500)**	**Tirilazad** **(*n* = 3552)**	**Utrecht** **(*n* = 433)**
Age – n (%)	11,931 (100)	117	228	1484	439	1500	3552	433
Year – mean (SD)	53 (13)	57 (9)	52 (13)	56 (13)	51 (15)	55 (15)	52 (13)	55 (13)
WFNS grade – n (%)	11,144 (93)	105 (90)	NR	1483	NR (100)	1431 (95)	3551 (100)	427 (99)
I-III	8869 (80)	87 (83)	NR	1138 (77)	NR	854 (60)	2751 (77)	330 (77)
IV-V	2275 (20)	18 (17)	NR	345 (23)	NR	577 (40)	800 (23)	97 (23)
Premorbid hypertension – n (%)	7609 (64)	NR	226 (99)	207 (14)	439 (100)	1441 (96)	3481 (98)	NR
Yes (%)	2884 (38)	NR	85 (38)	57 (28)	162 (37)	696 (48)	1147 (33)	NR
CT Fisher grade – n (%)	9745 (82)	104 (94)	NR	207 (14)	312 (71)	1434 (96)	3529 (99)	NR
1	493 (5)	3 (3)	NR	1 (1)	19 (6)	206 (14)	74 (2)	NR
2	2050 (21)	45 (43)	NR	22 (11)	79 (25)	315 (22)	756 (21)	NR
3	3762 (39)	39 (38)	NR	43 (21)	182 (58)	695 (49)	1046 (30)	NR
4	3440 (35)	17 (16)	NR	141 (68)	32 (10)	218 (15)	1653 (47)	NR
Aneurysm size – n (%)	8719 (73)	NR	228 (100)	159 (11)	435 (99)	1176 (78)	3522 (99)	NR
< 13 mm	7314 (84)	NR	214 (94)	143 (90)	331 (76)	1020 (87)	2594 (74)	NR
≥ 13 mm	1405 (16)	NR	14 (6)	16 (10)	104 (24)	156 (13)	928 (26)	NR
Aneurysm location – n (%)	9980 (90)	NR	228 (100)	457 (31)	437 (100)	1224 (75)	3487 (98)	389 (90)
ACA	1952 (20)	NR	13 (6)	6 (1)	14 (3)	63 (5)	1256 (36)	3 (1)
ACOM	2020 (20)	NR	80 (35)	184 (40)	119 (27)	332 (27)	0	173 (45)
ICA	1962 (20)	NR	36 (16)	38 (8)	46 (11)	134 (11)	1046 (30)	32 (8)
MCA	1840 (18)	NR	22 (10)	89 (20)	88 (20)	209 (17)	711 (20)	85 (22)
PCOM	899 (9)	NR	54 (24)	79 (17)	87 (20)	268 (22)	0	68 (18)
Posterior	1307 (13)	NR	23 (10)	61 (13)	83 (19)	218 (18)	475 (14)	28 (7)
Functional outcome – n (%)	10,653 (89)	109 (93)	207 (91)	1481 (100)	435 (99)	1151 (77)	3498 (98)	433 (100)
Favorable	8537 (80)	87 (80)	190 (92)	1083 (73)	333 (77)	755 (66)	2861 (82)	381 (88)
Unfavorable	2216 (20)	22 (20)	17 (8)	398 (27)	102 (23)	396 (34)	637 (18)	52 (12)

*Abbreviations*: *SD* Standard deviation, *ACA* Anterior cerebral artery, *ACOM* Anterior communicating aneurysm, *ICA* Internal carotid artery, *MCA* Middle cerebral artery, *PCOM* Posterior communicating artery, *Posterior* posterior circulation, *mm* Millimeter

^a^Full cohort: The randomized controlled trials are the Clazosentan to Overcome Neurological Ischemia and Infarction occurring after SAH trial (CONSCIOUS 1, *n* = 413), the Acute Systemic Erythropoietin Therapy to Reduce Delayed Ischemic Deficits following SAH, and the Effects of Acute Treatment with Statins on Cerebral Autoregulation in patients after SAH trials (EPO/Statin, *n* = 160); Heinrich Heine University Concomitant Intraventricular Fibrinolysis and Low-Frequency Rotation After Severe Subarachnoid Haemorrhage trial (HHU, n = 60); Intraoperative Hypothermia for Aneurysm Surgery Trial (IHAST, *n* = 1000); International Subarachnoid Aneurysm Trial (ISAT, *n* = 2143); Matrix and platinum science trials (MAPS, *n* = 228); the Magnesium Sulphate in Aneurysmal Subarachnoid Haemorrhage trials (MASH, n = 1484); and the Tirilazad trials (*n* = 3552). The observational registries are the SAH registry of the University of Chicago (*n* = 75); the University of Leeds Neurocognitive observation; the database of subarachnoid treatment of the University of Washington (D-SAT,* n* = 439); the subarachnoid haemorrhage outcomes project of Columbia University (SHOP, *n* = 1500); and the University Medical Center Utrecht registry (UMCU, *n* = 433)

### Cluster-specific baseline characteristics

We observed large heterogeneity in baseline characteristics between individual clusters (between-study heterogeneity). The proportion of patients presenting in unfavorable clinical condition (WFNS IV-V) ranged from 0% (IHAST) to 78% (HHU) and the proportion of aneurysms larger than 13 mm from 6% (MAPS) to 26% (Tirilazad). In the Tirilazad cluster and ISAT clusters, no posterior communicating artery aneurysms (PCOM) were included, in the Tirilazad cluster no anterior communicating artery aneurysms (ACOM), and in EPO/Statin no anterior cerebral artery aneurysms. Conversely, in the Conscious-I, EPO/Statin, I-HAST, MAPS, MASH1/2, and Utrecht clusters the majority (> 50%) of aneurysms were located at the ACOM and PCOM sites. Both the mean age and the proportion of hypertension were similar across clusters. Favorable functional outcome ranged from 60% (Chicago) to 92% (MAPS).

### Discrimination

The predictor effects were described in Supplementary Material 6. The internally validated, optimism-corrected Harrell’s *c*-statistic was 0.77 (95% CI 0.76–0.79, Table 2). We observed a large variability in discriminative performance when evaluating the *c*-statistics at single-study external validation. Model discrimination ranged from (very) poor, comparable to a coin flip, in the IMASH cluster (0.52, 95% CI 0.45–0.59), to moderate in the Leeds cluster (0.66, 95% CI 0.52–0.79), and the ISAT clusters (0.68, 95% CI 0.65–0.71), to excellent in the Conscious-1 (0.78, 95% CI 0.72–0.83), the D-SAT (0.80, 95% CI 0.75–0.85), and the SHOP clusters (0.84, 95% CI 0.82–0.86). The pooled mean *c*-statistic with cross-validation was 0.75 (95% CI 0.70–0.78). The I^2^-statistic was 0.92, indicating that the proportion of the total variability in the *c*-statistic was to a large extent explained by between-study heterogeneity.

**Table 2:** Model performance across validation methods

Validation technique	Performance metric			
	**Discrimination**		**Calibration**	
	***c*-statistic** **(95% CI)**	**Model-based c-statistic (95% CI)**	**Intercept** **(95% CI)**	**Slope** **(95% CI)**
Optimism-corrected *c*-statistic^a^	0.77 (0.76, 0.78)	NA	NI	NI
Single-study external validation^b^				
Conscious-1	0.78 (0.72, 0.83)	0.76	-0.10 (-0.36, 0.15)	1.12 (0.83, 1.38)
Chicago	0.73 (0.61, 0.84)	0.77	0.75 (0.23, 1.27)	0.82 (0.31, 1.34)
EPO/Statin	0.76 (0.68, 0.84)	0.76	0.55 (0.19, 0.91)	1.02 (0.64, 1.40)
HHU	0.75 (0.59, 0.91)	0.71	-1.40 (-2.08, -0.71)	1.17 (0.16, 2.17)
IHAST	0.71 (0.66, 0.76)	0.68	0.02 (-0.17, 0.21)	1.21 (0.91, 1.51)
IMASH	0.52 (0.45, 0.59)	0.78	0.42 (0.16, 0.67)	0.10 (-0.11, 0.30)
ISAT	0.68 (0.65, 0.71)	0.70	-0.02 (-0.15, 0.11)	0.96 (0.80, 1.12)
Leeds	0.66 (0.52, 0.79)	0.76	-0.02 (-0.52, 0.48)	0.53 (0.05, 1.01)
MAPS	0.81 (0.70, 0.92)	0.74	-0.91 (-1.38, -0.44)	1.25 (0.76, 1.73)
MASH1/2	0.76 (0.73, 0.79)	0.77	0.25 (0.12, 0.37)	0.99 (0.86, 1.12)
D-SAT	0.80 (0.75, 0.85)	0.78	0.21 (-0.04, 0.45)	1.24 (0.96, 1.51)
SHOP	0.84 (0.82, 0.86)	0.80	0.47 (0.35, 0.59)	1.31 (1.18, 1.45)
Tirilazad	0.76 (0.74, 0.78)	0.77	-0.38 (-0.48, -0.29)	0.91 (0.82, 1.00)
Utrecht	0.76 (0.68, 0.83)	0.78	-1.03 (-1.34, -0.72)	0.89 (0.60, 1.17)
Leave-one-cluster-out cross-validation^b^				
Mean (95% CI) – I^2^	0.74 (0.70, 0.78) – 92	NI	-0.06 (-0.37, 0.24) – 97	0.96 (0.78, 1.13) – 90
Range	0.52–0.84	NI	-1.40–0.75	0.53–1.31

*Abbreviations*: *CI* confidence interval, *NI* not informative

^a^Optimism-corrected *c*-statistic computed with 200 bootstrap samples

^b^Model validated in the cluster left out at development. Pooled performance measures obtained with random effects meta-analysis

We observed a substantial decrease in discriminative performance in 6 clusters compared to the optimism-corrected *c*-statistic (benchmark) and the pooled mean c-statistic (reference for overall external performance). Specifically, in the Leeds, IMASH, and Chicago clusters, this decline can be attributed to case-mix variation (Harrell’s 0.66, 0.52, and 0.76 versus model-based 0.76, 0.78, and 0.73, respectively). In the HHU and IHAST clusters, the drop is due to miscalibration (Harrell’s 0.73 and 0.71 versus model-based 0.71 and 0.68, respectively) and in the ISAT cluster it is explained by a combination of case-mix variation and miscalibration (Harrell's 0.68 and model-based 0.70).

### Calibration

**Fig. 1:**
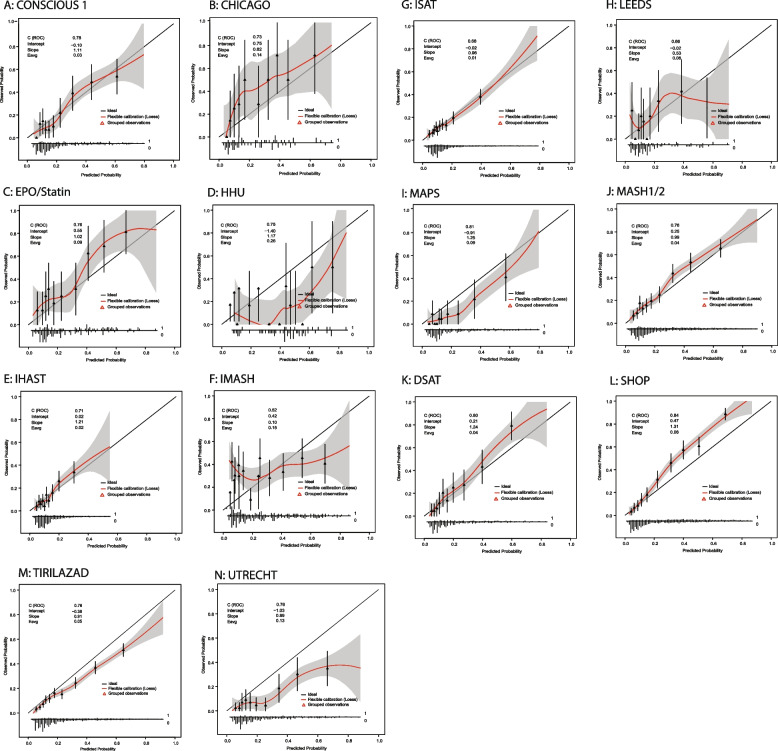
**A-M** Internal-External Cross-Validation Calibration Plots. Abbreviations: C (ROC) = *c*-statistic or receiving operating curve

Again, we observed large variability in the calibration with single-study external validation across clusters. Calibration ranged from poor in the HHU cluster with an intercept of -1.40 (95% CI -2.08–-0.71, Fig. 1A-N) and slope of 1.17 (0.16–2.17) to excellent in the CONSCIOUS-I, IHAST, ISAT, MASH1/2, and D-SAT clusters. The pooled mean intercept was -0.06 (95% CI -0.37–0.24) and the pooled mean slope was 0.96 (95% CI 0.78–1.17) with cross-validation. For both the intercept (I^2^ = 0.97) and the slope (I^2^ = 0.90), the I^2^-statistic indicated that the degree of total variability was largely explained by between-study heterogeneity.

### Transportability

In most clusters the membership models’ *c*-statistics were moderately high (IHAST, IMASH, ISAT, MAPS, DSAT, SHOP, and Utrecht, all between 0.70–0.80, Table 3) or high (CONSCIOUS-I, Chicago, EPO/Statin, HHU, and Tirilazad, all above 0.80). Despite this, the discriminative performance remained satisfactory indicating good transportability of the model. In other words: even in distinctly different study populations the model discriminated well between high-risk and low-risk patients. There was one exception to this generalization. In the Leeds cluster the derivation and validation samples were similar (membership model *c*-statistic 0.69), but the discriminative performance was unsatisfactory indicating poor generalizability of the model (Supplementary Material 7).

**Table 3:** Relatedness of derivation versus validation cohorts with internal–external cross-validation clusters assessed by the membership model

Cluster	*c*-statistics of the membership model	*c*-statistics at derivation (95% CI)
Conscious 1	0.80	0.78 (0.72–0.83)
Chicago	0.85	0.72 (0.61–0.84)
EPO/Statin	0.84	0.75 (0.68–0.84)
HHU	0.93	0.74 (0.59–0.91)
IHAST	0.76	0.71 (0.66–0.76)
IMASH	0.79	0.53 (0.45–0.59)
ISAT	0.70	0.68 (0.65–0.71)
Leeds	0.69	0.65 (0.52–0.79)
MAPS	0.73	0.75 (0.70–0.92)
MASH1/2	0.62	0.78 (0.73–0.79)
DSAT	0.73	0.79 (0.75–0.85)
SHOP	0.78	0.84 (0.74–0.86)
Tirilazad	0.82	0.76 (0.74–0.78)
Utrecht	0.74	0.75 (0.69–0.83)

The membership model assesses the outcome “membership of development set” (1) or “membership of validation set” (0) as a function of functional outcome and the predictors of functional outcome. The *c*-statistic represents the degree of relatedness between the development set and the validation set. If two datasets are very different this will result in easier (higher) discrimination and vice versa. Thus, a high *c*-statistic means low relatedness, and a low *c*-statistic high relatedness. The higher the relatedness the more the performance represents reproducibility (or generalizability). The lower the relatedness the more the performance could represent transportability

## Discussion

We used the SAHIT data repository, a large individual participant meta-analysis dataset, to study external validity in a large number of single-study cohorts to illustrate the potential pitfalls in interpreting model performance, of single-study external validation. Although single-study external validation is preferred over no at all, our analysis clearly illustrates two pitfalls and how this may lead to a false appreciation of the model.

(1) We observed that model performance was highly variable between cohorts. This means that the appreciation of the model is highly dependent on the choice of the validation data. For example, validating the model in the IMASH (0.52), Leeds (0.66), or the ISAT cluster (0.68) would indicate poor to moderate discrimination. Contrarily, the reference mean *c*-statistic (0.74) and most cluster-specific *c*-statistics suggest otherwise. There is no formal threshold for the *c*-statistic for implementation in clinical practice. Conversely, the model performance in the SHOP cluster (0.84) was probably more optimistic than can be expected in the population. A similar problem is observed when examining the calibration of the model at external validation. In some clusters such as HHU, I-MASH, Leeds or SHOP calibration was (very) poor, but in others it was excellent. Several factors may explain the large variation in performance.

First, case-mix variation is known to influence discriminative performance [39, 40]. The more patients within a cluster are alike (homogeneous) the more difficult it is to discriminate between high and low-risk individuals. It is known that ISAT excluded 90% of initially screened patients leaving patients with similar characteristics such as favorable prognosis and aneurysms predominantly located at specific sites [41]. As such, in ISAT we observed a slightly higher model-based *c*-statistic than Harrell’s *c*-statistic. Validating the model in the ISAT cohort alone may lead to a false conclusion about the model’s discriminative performance.

Second, between-study heterogeneity can also affect model performance. Case-mix variation refers to the differences between subjects within a population, while between-study heterogeneity refers to differences between study populations. Slight changes in the definition or the measurement of predictors and the outcome can change the size and direction of predictor effects [42]. Additionally, predictors may affect treatment decisions downstream (confounding by indication) and subsequently affect patient outcomes. Because of this, such slight changes can lead to severe miscalibration and poor discrimination of a model.

With cross-validation, between-study heterogeneity can be used to benefit interpretation. The SAHIT registry consists of data from randomized trials that have had stringently selected (thus homogeneous) study populations, that together, form a very heterogeneous overall study population. Differences in predictor and outcome definition or measurement and the context of the study period and geographical origin of the SAHIT registry can be used to explain variability in model performance. The I^2^-statistics obtained with random effects meta-analysis confirmed that the proportion of total variability in model performance was largely explained by between-study heterogeneity.

(2) Without understanding the heterogeneity between the derivation and validation sample and the population we cannot assess the generalizability (i.e., reproducibility in a similar population) and the transportability (i.e., reproducibility in a different but related population) of the model [43, 44]. For example, the intended subpopulation – in which the model is to be applied – may have distinct differences from the validation sample (e.g., in the healthcare setting, measurement of biomarkers or imaging findings, and treatment algorithms). Also, the validation may have been conducted in an independent, but equally selected sample not representative of the population. In both cases, we do not obtain a valid insight into the expected model performance.

Due to overall improvements in diagnostic capabilities, treatment algorithms, and patients’ outcomes the validity of all models will eventually expire. Thus, even if a model performs well it will most likely not be globally and eternally applicable [45]. Because of this, most models will need local and continuous updating. Some clinical prediction models will require updating of the intercept and/or the slope, while others may need a full re-estimation of the parameters. Geographical, temporal, or methodological clustering can be utilized to assess model performance across multiple dimensions and inform updating efforts to fit the intended context. In addition, the membership model can be used to obtain a gross estimate of the relatedness of two samples.

### Strengths and Limitations

This study is strengthened by the use of a large individual participant meta-analysis dataset to assess model performance across multiple dimensions. Furthermore, because of the clustered nature of the SAHIT data repository, we were able to investigate between-cluster heterogeneity, generalizability, and transportability.

Our study is limited by the fact that in some clusters predictors were missing completely. We performed multiple imputation of the entire SAHIT data repository instead of the individual clusters. This may mean that between-cluster heterogeneity could be diluted and that model performance may be overestimated for individual clusters with completely missing predictor variables. The largest proportion of missingness for the full cohort was with premorbid hypertension (36%) and will not have a large effect on the overall conclusions of this study.

Another limitation of our study was the use of multiple outcome scales and varying time points of assessment. We chose to use the GOS at 3 months as much as possible, but not all studies assessed outcome equally. Patients can improve or worsen from 3 months up to 12 months, but we hypothesize that even though on an ordinal scale these changes will be substantial on a dichotomized scale this might be limited.

### Recommendations

Insight into case-mix variation, between-cluster heterogeneity, generalizability, and transportability is required to decide if a clinical prediction model performs well enough for to be considered for implementation. Implementation without these insights could lead to patient harm due to inaccurate medical decision-making and possibly incentivize the development of new clinical prediction models instead of validating already existing ones, thereby contributing to research waste. Even for a relatively rare disease an abundance of models predicting functional outcome in aSAH patients already exist [46–50]. Most of these models contain more or less the same set of predictors. The rising availability of clustered data from large international collaborations will open up possibilities for leave-one-cluster-out cross-validation and should be utilized [51].

We advocate using cross-validation instead of single-study external validation, but there are also disadvantages to this approach. First, cross-validation requires a large clustered dataset with sufficient patients per cluster that is usually obtained through international collaborations and may not always be available. Second, an important feature of external validation is an independent evaluation of a clinical prediction model. Usually, leave-one-cluster-out internal–external cross-validation will be conducted directly after model development and thus not performed independently. To aid transparency, regression formulas, code, and data should be made publicly available.

If not available, a reasonable alternative strategy is conducting multiple (smaller) single-study external validations each exploring another dimension. We stress that a single single-study external validation cannot be interpreted as decisive proof of a well or poor model performance and that local and continuous validation is usually required. As a minimum, we advise evaluating selection criteria, recruitment dates, geographical location, and study design of the development and the validation data to obtain a gross estimate of between-cluster heterogeneity.

## Conclusions

Two potential pitfalls of single-study external validation have to be considered when interpreting such a validation study. (1) Model performance with single-study external validation can depend heavily on the choice of validation data and can thus lead to a false appreciation of a clinical prediction model. (2) To accurately appreciate generalizability and transportability it is necessary to investigate heterogeneity between the derivation and validation data and the representativeness to the intended population. Thus, a single validation is not equipped to draw definitive conclusions about model performance. As an alternative leave-one-cluster-out internal–external cross-validation enables inspecting model performance across multiple settings with varying temporal, geographical, and methodological dimensions and can inform more reliably about expected performance and whether local revision is required.

### Supplementary Information

Supplementary Material 1.

## Data Availability

We are not at liberty to make the data supporting this publication available. Patient-level data is available upon reasonable request to the SAHIT data repository. More detailed information about the SAHIT data repository including an ethical statement is available elsewhere [52, 53]. Upon publication, Rcode will be made publicly available via: https://github.com/WinkelJordi/crossvalidation.

## Abbreviations

(a)SAH: Aneurysmal subarachnoid hemorrhage
CT: Computed tomography
GOS: Glasgow Outcome Scale
mRS: Modified Rankin Scale
RCT: Randomized controlled trial
SAHIT: Subarachnoid Haemorrhage International Trialists’
WFNS: World Federation of Neurological Surgeons grade
