# Supplementary material for: Pitfalls of single-study external validation illustrated with a model predicting functional outcome after aneurysmal subarachnoid hemorrhage
Source: BMC Med Res Methodol. 2024 Aug 8;24:176. doi: 10.1186/s12874-024-02280-9 (PMC11308226; doi:10.1186/s12874-024-02280-9)
Supplement: Supplementary file 1 — Supplementary Material 1. [file 12874_2024_2280_MOESM1_ESM.pdf]

Supplementary Materials

Supplementary Material 1. SAHIT data repository study characteristics.

Supplementary Material 2. Excluded studies and the reason for exclusion.

Supplementary Material 3. Outcome imputation algorithm.

Supplementary Material 4. Explanations of performance measures discussed in this study.

Supplementary Material 5. TRIPOD Statement for prediction model validation.

Supplementary Material 6. Predictor effect of the multivariable model.

Supplementary Material 7. Conceptual framework of evaluating Harrell's *c*-statistic and the membership models *c*-statistic.

Supplementary Material 1. SAHIT data repository study characteristics.

Studies	Type	Study population	Enrolment	Outcome measures	Sample size
*Trials**					
ALISAH	Phase IB	Multicenter	2006-2010	Barthel Index, NIHSS, Stroke Impact Scale, GOS and mRS	47
BRANT	Phase III	Multicenter	1985-1987	GOS	554
CONSCIOUS-I	Phase II	Multicenter	2005-2006	GOSE	413
EPO/Statin	Phase II	NR	NR	GOS	160
HHU	Phase II	Single center	2008-2011	GOS, Clot clearance rate, DCI	60
IHAST	Phase III	Multicenter	2000-2003	GOSE	1001
I-MASH	Phase III	Multicenter	2002-2008	GOSE	327
ISAT	Phase III	Multicenter	1997-2002	mRS	2143
MAPS	Phase IV	Multicenter	2007-2011	TAR and mRS	228
MASH-I/II	Phase II/III	Multicenter	2000-2011	DCI and mRS	1484
Tirilazad	Phase III	Multicenter	1991-1997	GOS	3552
*Registries***					
CARAT	Ambi-directional hospital registry	Multicenter	1996-1998	Non-procedural rerupture	1010
Chicago	Prospective hospital registry	Single center	1995-2002	mRS	75
D-SAT	Prospective hospital registry	Single center	1983-1993	GOS, mRS, Barthel, NIHSS	439

Durham	Prospective hospital registry	Single center	NR	Neurocognitive battery	105
Kurashiki	Hospital registry	Single center	NR	NR	NR
Leeds	Prospective hospital registry	Single center	2005-2006	mRS, Neurocognitive battery	117
SHOP	Prospective hospital registry	Single center	1996-2012	GOS, mRS, Barthel, NIHSS	1500
St. Michael's	Hospital registry	Single-center	NR	NR	173
SWISS	Nationwide registry	Multicenter	NR	NR	NR
UMCU	hospital registry	Single-center	NR	NR	433

Abbreviations: DCI = delayed cerebral ischemia; mRS = modified Rankin Scale; GOS(E) = Glasgow Outcome Scale (Extended); NIHSS = National Institutes of Health Stroke Scale; NR = not reported; TAR = target aneurysm recurrence.

* ALISAH = Albumin in Subarachnoid Hemorrhage Trial; BRANT = the British Aneurysm Nimodipine trial; CONSCIOUS-I = The randomized controlled trials are the Clazosentan to Overcome Neurological Ischemia and Infarction occurring after SAH trial; EPO/Statin = the Acute Systemic Erythropoietin Therapy to Reduce Delayed Ischemic Deficits following SAH, and the Effects of Acute Treatment with Statins on Cerebral Autoregulation in patients after SAH trials; HHU = Heinrich Heine University Concomitant Intraventricular Fibrinolysis and Low-Frequency Rotation After Severe Subarachnoid Haemorrhage trial; IHAST = Intraoperative Hypothermia for Aneurysm Surgery Trial; I-MASH = Intravenous Magnesium Sulphate for Aneurysmal Subarachnoid Haemorrhage trial; ISAT = International Subarachnoid Aneurysm Trial; MAPS = Matrix and platinum science trials; the Tirilazad trials; MASH-I = the Magnesium Sulphate in Aneurysmal Subarachnoid Haemorrhage trials

** CARAT = cerebral aneurysm re-rupture after treatment; the SAH registry of the University of Chicago; D-SAT = the dataset of subarachnoid treatment of the University of Washington;

Observational Neurocognitive Study from University of Durham, Durham, United Kingdom; the Hospital registry from Kurashiki Central Hospital, Japan; University of Leeds Neurocognitive observation, Leeds, United Kingdom, SHOP = the subarachnoid haemorrhage outcomes project of Columbia University; St Michael's Hospital, Toronto, Canada; the Swiss study on SAH—a nationwide registry of SAH from Switzerland (SWISS); University Medical Centre Utrecht SAH Registry, Utrecht, The Netherlands.

Supplementary Material 2. Excluded studies and the reason for exclusion.

**Study**	**Reason for exclusion**
ALISAH	No data available
BRANT	No data available
CARAT	No outcome data available
Durham	No outcome data available
I-MASH	No outcome data available
Kurashiki	No data available
St. Michael's Hospital	No data available
SWISS	No data available

ALISAH = Albumin in Subarachnoid Hemorrhage Trial; BRANT = the British Aneurysm Nimodipine trial; CARAT = cerebral aneurysm re-rupture after treatment; Observational Neurocognitive Study from University of Durham, Durham, United Kingdom; I-MASH = Intravenous Magnesium Sulphate for Aneurysmal Subarachnoid Haemorrhage trial; the Hospital registry from Kurashiki Central Hospital, Japan; St Michael's Hospital, Toronto, Canada; the Swiss study on SAH—a nationwide registry of SAH from Switzerland (SWISS).

Supplementary Material 3. Outcome imputation algorithm.

GOS	Elaboration	eGOS		mRS	
5	Good recovery	8	Upper good recovery	0	No symptoms
		7	Lower good recovery	1	No significant disability despite symptoms able to carry out all usual duties and activities
		6	Upper moderate disability		
4	Moderate disability	5	Lower moderate disability	2	Slight disability; unable to carry out all previous activities
		4	Upper severe disability	3	Moderate disability requiring some help
3	Severe disability	3	Lower severe disability	4	Moderate severe disability; unable to walk and attend to bodily needs
2	Vegetative state	2	Vegetative state	5	Severe disability; bedridden
1	Dead	1	Dead	6	Dead

Supplementary Material – Table 4. Explanations of performance measures discussed in this study.

**Measure**	**Explanation**
Model performance	
Discrimination	The ability to discriminate between high-risk patients and low-risk patients. Does the model accurately identify those that experience the outcome versus those that do not?
Harrell's *c*-statistic	Used to assess discriminative performance for a binary outcome. The *c*-statistic is the proportion of all possible pairs of observations discordant with the outcome (i.e., one with the outcome and one without), in which the subject with the outcome had a higher predicted probability than the one without the outcome. A *c*-statistic of 0.5 means an uninformative model and a c-statistic of 1 means perfect discrimination.
Optimism-corrected *c*-statistic	Obtained through bootstrap validation. The difference between apparent and optimism-corrected c-statistic is called optimism. Optimism is the overestimation of the predictive performance due to modelling random noise in the development data.
Model-based c-statistic	Case-mix heterogeneity-controlled measure of discriminative performance. The model-based c-statistic assumes the coefficient are correct. The differences between Harrell's *c*-statistic and the model-based *c*-statistic can be interpreted as the difference due to case-mix variation.
Calibration	The agreement between the predicted risk and the observed risk. How accurate are the risk predictions?
Calibration slope	The intercept is the ratio between expected and observed outcomes. Ideally, the intercept has a value of 0, whereas a negative value means an overestimation of the predicted risk to the observed risk, and a positive value is an underestimation of the predicted risk to the observed risk.
Calibration intercept	The beta value of the calibration model. Evaluates the spread of the estimated risks and ideally have a value of 1. A value below

	1 means the estimated risks are too extreme and a value above 1 means the estimated risks are too moderate.
Eavg	Average absolute difference in the predicted and observed probabilities.

Supplementary Material 5. TRIPOD Statement for prediction model validation.

Section/Topic	ı	Checklist Item	Page
**Title and abstract**			
Title	1	Identify the study as developing and/or validating a multivariable prediction model, the target population, and the outcome to be predicted.	Title page
Abstract	2	Provide a summary of objectives, study design, setting, participants, sample size, predictors, outcome, statistical analysis, results, and conclusions.	Abstr page
**Introduction**			
Background and objectives	3a	Explain the medical context (including whether diagnostic or prognostic) and rationale for developing or validating the multivariable prediction model, including references to existing models.	3-4
	3b	Specify the objectives, including whether the study describes the development or validation of the model or both.	4
**Methods**			
Source of data	4a	Describe the study design or source of data (e.g., randomized trial, cohort, or registry data), separately for the development and validation data sets, if applicable.	4
	4b	Specify the key study dates, including start of accrual; end of accrual; and, if applicable, end of follow-up.	Suppl
Participants	5a	Specify key elements of the study setting (e.g., primary care, secondary care, general population) including number and location of centres.	Suppl
	5b	Describe eligibility criteria for participants.	Suppl
	5c	Give details of treatments received, if relevant.	NA
Outcome	6a	Clearly define the outcome that is predicted by the prediction model, including how and when assessed.	5
	6b	Report any actions to blind assessment of the outcome to be predicted.	NA
Predictors	7a	Clearly define all predictors used in developing or validating the multivariable prediction model, including how and when they were measured.	4-5
	7b	Report any actions to blind assessment of predictors for the outcome and other predictors.	NA
Sample size	8	Explain how the study size was arrived at.	4
Missing data	9	Describe how missing data were handled (e.g., complete-case analysis, single imputation, multiple imputation) with details of any imputation method.	5
Statistical analysis methods	10c	For validation, describe how the predictions were calculated.	5
	10d	Specify all measures used to assess model performance and, if relevant, to compare multiple models.	5
	10e	Describe any model updating (e.g., recalibration) arising from the validation, if done.	NA
Risk groups	11	Provide details on how risk groups were created, if done.	NA
Development vs. validation	12	For validation, identify any differences from the development data in setting, eligibility criteria, outcome, and predictors.	7-8
**Results**			
Participants	13a	Describe the flow of participants through the study, including the number of participants with and without the outcome and, if applicable, a summary of the follow-up time. A diagram may be helpful.	4-5
	13b	Describe the characteristics of the participants (basic demographics, clinical features, available predictors), including the number of participants with missing data for predictors and outcome.	7-8, Table 1
	13c	For validation, show a comparison with the development data of the distribution of important variables (demographics, predictors and outcome).	Table 1
Model performance	16	Report performance measures (with CIs) for the prediction model.	8-9
Model-updating	17	If done, report the results from any model updating (i.e., model specification, model performance).	NA
**Discussion**			
Limitations	18	Discuss any limitations of the study (such as nonrepresentative sample, few events per predictor, missing data).	12-13
Interpretation	19a	For validation, discuss the results with reference to performance in the development data, and any other validation data.	8-9, 10-12
	19b	Give an overall interpretation of the results, considering objectives, limitations, results from similar studies, and other relevant evidence.	10-14
Implications	20	Discuss the potential clinical use of the model and implications for future research.	NA

Other information			
Supplementary information	21	Provide information about the availability of supplementary resources, such as study protocol, Web calculator, and data sets.	4
Funding	22	Give the source of funding and the role of the funders for the present study.	Back matte r

Supplementary Material 6. Predictor effect of the multivariable model.

**Variable**	**Beta**	**Multivariable (OR-95% CI)**
Intercept	-3.8	NA
Age (per decade)	0.0	1.0 (0.9-1.1)
	0.4	1.5 (1.3-1.6)
Premorbid hypertension (yes)	0.4	1.6 (1.3-1.7)
WFNS grade		
I-III	Ref	Ref
IV-V	1.6	4.9 (4.4-5.5)
CT Fisher grade		
1	Ref	Ref
2	0.7	1.9 (1.3-3.0)
3	1.1	3.1 (2.0-4.6)
4	1.4	4.0 (2.7-5.9)
Aneurysm size		
< 13 mm	Ref	Ref
≥ 13 mm	0.6	1.8 (1.5-2.1)
Aneurysm location		
ACA	Ref	Ref
ACOM	0.3	1.4 (1.1-1.6)
ICA	0.1	1.1 (0.9-1.3)
MCA	-0.0	1.0 (0.8-1.2)
PCOM	0.3	1.3 (1.0-1.7)
Post	0.5	1.6 (1.4-1.9)

Abbreviations: ACA =, ACOM = anterior communicating aneurysm, ICA = internal carotid artery, MCA = middle cerebral artery, PCOM = posterior communicating artery, Post = posterior circulation, mm = millimeter.

Supplementary Material 7. Conceptual framework of evaluating Harrell's *c*-statistic and the membership models *c*-statistic.

		**Membership models' *c*-statistic**			
		High	Interpretation	Low	Interpretation
**Harrell's *c*-statistic**	High	↑↑	Population is distinctly different with satisfactory discrimination: transportable.	↑↓	Population is similar with satisfactory discrimination: reproducible.
	Low	↓↑	Population is distinctly different without satisfactory discrimination: not transportable.	↓↓	Population is similar without satisfactory discrimination: not reproducible.
